# A Rare Presentation of Direct Traumatic Optic Neuropathy in a Patient Poked in the Eye by an Antenna

**DOI:** 10.7759/cureus.18244

**Published:** 2021-09-24

**Authors:** Jake E Tenewitz, Evan J Chen, Mont J Cartwright

**Affiliations:** 1 Medicine, University of Central Florida College of Medicine, Orlando, USA; 2 Ophthalmology, Medical Eye Associates, Kissimmee, USA

**Keywords:** traumatic optic neuropathy, direct optic nerve injury, indirect optic nerve injury, antenna, acute vision loss

## Abstract

Traumatic optic neuropathy (TON) is defined as a loss of vision due to a traumatic injury to the optic nerve. Numerous mechanisms may contribute to the development of TON; however, most cases involve injuries to the globe, orbit, or adnexa. This case report presents a 24-year-old male who was inadvertently poked in the eye with an antenna and developed a direct optic nerve injury in the absence of a significant injury to the surrounding orbital structures. A CT scan was used to confirm the diagnosis. No visual recovery was observed throughout his clinical course. The proposed pathophysiologic mechanisms, clinical presentation, complications, treatments, and prognosis of traumatic optic neuropathy are subsequently discussed.

## Introduction

Traumatic optic neuropathy (TON) is a condition described by a pronounced loss of vision secondary to an acute, traumatic insult to the optic nerve. TON presents on a spectrum ranging from a contusion to complete transection with the latter resulting in complete, permanent vision loss in the affected eye. TON is a rare phenomenon because of the optic nerve’s location within the ocular socket and behind the globe. It is thus usually accompanied by orbital wall fractures or some form of globe luxation or damage. According to Kosaki et al., as many as 38.2% of patients with globe luxation also experience avulsion of the optic nerve [1].

Optic nerve injuries can be categorized as primary or secondary. Primary damage involves an external traumatic force that leads to injury of nerve fibers or nerve vessels, and thus immediate loss of vision [2]. Secondary damage involves disruption of the optic nerve blood supply, leading to ischemia without immediate loss of vision [2].

TON can be classified as direct or indirect. Direct TON occurs when the optic nerve is directly injured via a penetrating (foreign body) or shearing (orbital fracture) mechanism. Indirect TON occurs when the force of blunt head trauma is transmitted to the optic nerve often via the optic canal. Motor vehicle accidents are the most common causes of TON [3]. Indirect TON has been reported in up to 0.5-5% of closed head injuries [4].

This report presents a patient who developed a direct optic nerve injury upon being inadvertently poked in the eye by an antenna. The injury occurred in the absence of a full-thickness lid laceration or insult to the globe, orbit, or adnexa. Despite the lack of lid perforation, a direct optic nerve injury is proposed whereby the antenna traumatized the fragile optic nerve in the intraorbital segment.

## Case presentation

A 24-year-old male was bending down to pick up an object off the garage floor when he was inadvertently poked by an antenna on his motorcycle in the right lower lid (RLL). The patient reported an immediate, complete visual loss in the right eye accompanied by severe ocular pain. He subsequently presented to a local emergency department with no light perception (NLP) vision in the right eye and a 0.5cm superficial circular puncture wound in the RLL. Non-contrast CT of the orbits was acquired and initially read as unremarkable. He received pain management and wound care for the laceration. He was instructed to follow up with an outpatient ophthalmologist.

**Figure 1:**
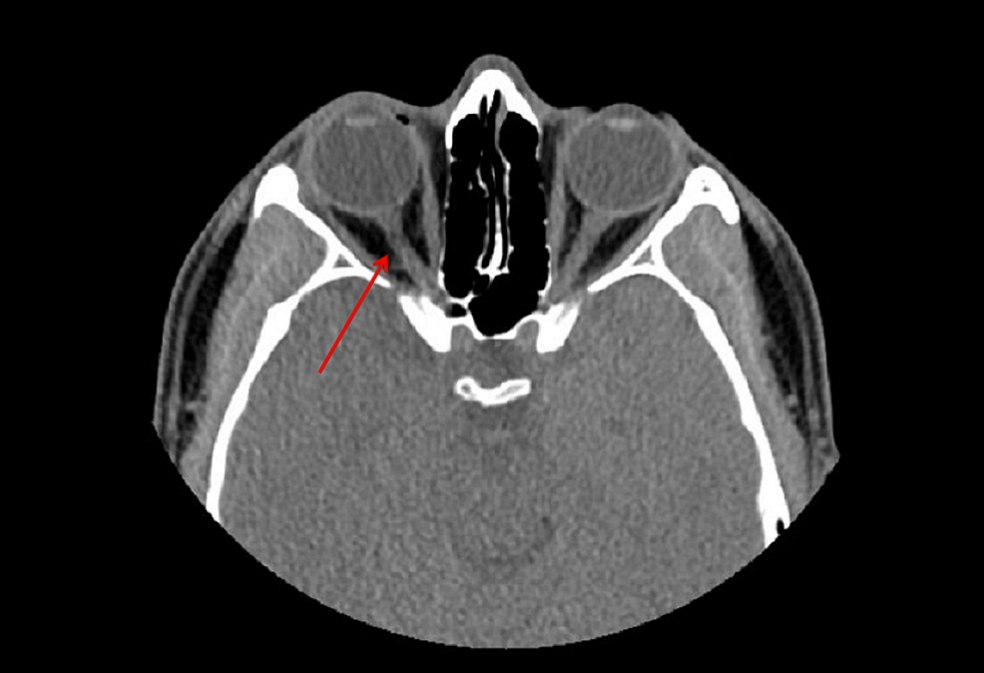
Non-contrast CT of the orbits demonstrating possible incomplete transection or compression injury of the right intraorbital optic nerve in the absence of orbital wall fractures, globe damage, or luxation.

The patient presented to an ophthalmologist (MJC) the next morning with no subjective improvement in vision and persistent ocular pain. The best-corrected visual acuity was NLP in the right eye (*oculus dexter* (OD)) and 20/25 in the left eye (*oculus sinister* (OS)). A 2+ relative afferent pupillary defect (RAPD) was observed in the right eye. Extraocular movements and alignment were intact in both eyes. Intraocular pressures (IOP) were 16mmHg OD and 17mmHg OS. All exam findings in the left eye were within normal limits. A slit lamp exam of the right eye demonstrated a medial laceration of the RLL 5-7mm from the margin and right upper lid (RUL) ptosis. The laceration in the RLL was superficial; it did not penetrate full thickness through the RLL. A slit lamp exam of the right eye was otherwise unremarkable. Importantly, the conjunctiva was clear and quiet and there was no indication of globe injury. Funduscopic exam of the right eye was unremarkable. There were no peripapillary or vitreous hemorrhages and the disk margins were clear with no swelling or pallor. The initial non-contrast CT of the orbits was re-read by the ophthalmologist with the following findings: possible incomplete transection or compression injury of the right intraorbital optic nerve in the absence of orbital wall fractures and globe damage or luxation (Figure 1). Based on the imaging and clinical findings, a direct optic nerve injury was suspected. An MRI was advised but not obtained due to the patient's financial situation. This would have provided further diagnostic clarity. The patient was managed with observation. Treatment options such as corticosteroids or optic nerve decompression were not pursued after discussion of their risks, benefits, and effectiveness with the patient.

On follow-up, two weeks status post injury, visual acuity of the right eye showed no improvement, and pain elicited upon abduction of the right eye persisted. Funduscopic exam revealed the development of right optic disk pallor in the temporal region with no associated edema or hemorrhage. At six weeks status post injury, the right optic nerve was diffusely pale, and visual acuity remained NLP. Resolution of the ocular pain and RUL ptosis was observed. The exam was unchanged at four months. The patient was subsequently scheduled for an annual eye exam to monitor for post-traumatic ocular pain and routine care for his left (monocular) eye. 

The patient had no past ocular or medical history. Prior to the trauma, his best-corrected visual acuity was 20/20 bilaterally. Family and social histories were non-contributory.

## Discussion

This patient had a classic presentation of TON upon being poked in the eye by the antenna: acute vision loss to NLP, relative afferent pupillary defect, and normal slit lamp exam save the RLL superficial lid laceration and RUL ptosis. CT imaging revealed a possible incomplete transection or compression injury of the right intraorbital optic nerve thus confirming the diagnosis. MRI imaging would have allowed for a better characterization of the lesion but it wasn’t obtained due to the patient’s financial situation. While the lesion was difficult to characterize on CT imaging, it was nonetheless significant and readily apparent. 

Despite no signs of globe injury/luxation, orbital wall fractures, or full-thickness lid lacerations, all of which would suggest a penetrating etiology, it is likely that the antenna penetrated the ocular socket medially and compromised the optic nerve. This “penetrating” mechanism of injury in the absence of a full-thickness laceration to the eyelid is possible given the relatively short course from the medial surface of the eye to the optic nerve, and the extreme elasticity of eyelids. The medial location of the partial-thickness laceration on the RLL supports this seemingly unlikely mechanism of injury. Furthermore, the optic nerve is extremely fragile and the force of impact from the antenna head, even though the intact eyelid, was sufficient to disrupt the nerve fibers.

This case thus represents a direct optic nerve injury, which is most commonly induced by a penetrating mechanism but can occur in the setting of an orbital wall fracture [5]. The location of the injury in the intraorbital segment strengthens the argument for a direct optic nerve injury as this would be the segment of nerve first encountered by a penetrating foreign body in the orbit. In comparison to indirect optic nerve injury, direct optic nerve injury carries a worse prognosis. Nishi et al. presented a case of indirect TON in which an isolated optic nerve injury was caused by a blunt injury to the inferior orbital rim without the involvement of the globe, leading to immediate and complete visual loss of the eye [6]. While the mechanism of injury was different, the initial clinical presentation was similar. However, their patient experienced moderate visual recovery and optic disc pallor at three months post injury while our patient did not experience any visual recovery and had optic disc pallor at two weeks post injury. It is unlikely that the antenna impacted the inferior orbital rim and generated a sufficient force to cause an indirect TON. 

Optic nerve damage can be associated with trauma to the globe. When the globe is struck, a sudden drastic increase in IOP can result in avulsion of the optic nerve. The shearing of the nerve from the globe occurs at the lamina cribrosa as it is a site of anatomic weakness due to lack of supportive connective tissue. Additionally, trauma to the globe can induce sudden retropulsion followed by transient anterior translocation, due to increased intraorbital pressure, leading to optic nerve avulsion [5]. More profound trauma can cause subluxation of the globe and resulting avulsion or transection. It is possible that the tip of the antenna struck the globe through the lower lid and damaged the optic nerve as discussed above. However, there were no signs of injury to the globe on clinical exam or CT imaging and thus this scenario is less likely. Furthermore, signs of optic nerve avulsion were not seen on exam or CT imaging. 

Intrusion of a foreign body into the orbit can cause TON via direct injury to the nerve or globe as discussed above. In addition, foreign body intrusion into the orbit can cause extreme rotation of the globe or anterior subluxation, both of which can cause optic nerve avulsion [7,8]. These mechanisms are possible but less likely as neither optic nerve avulsion or subluxation/displacement of the globe were seen on exam or CT imaging.

Management of patients with direct or indirect TON remains controversial. The landmark International Optic Nerve Trauma Study found that there was no benefit from either corticosteroid therapy or optic canal decompression surgery relative to observation [9]. However, a study by Chou et al. found that corticosteroid therapy or optic nerve decompression surgery resulted in significant visual recovery relative to observation [10]. More recently, a meta-analysis conducted by Wladis et al. found that there was no consistent benefit for any interventions in the management of TON [11]. The authors manage TON on a case by case basis taking into consideration the entire clinical picture. This patient who presented with NLP vision and CT findings concerning for direct optic nerve injury was managed with observation. His vision did not improve through the course of follow-up.

## Conclusions

Several cases of TON associated with trauma to the globe, orbit, or adnexa have been reported in the literature. This case is unique in that the suspected optic nerve injury occurred in the absence of significant trauma to the globe, orbit, or adnexa. The only sign of injury to our patient was a superficial RLL laceration. The mechanism of injury whereby the antenna compromised the friable optic nerve through the intact lower lid is quite peculiar. 

This patient who presented with a direct TON confirmed on CT imaging and NLP vision, did not experience any visual recovery. Injury to the optic nerve carries a poor prognosis in visual recovery with NLP vision and orbital wall fractures being associated with a worse prognosis. The vision loss may be permanent or may demonstrate gradual partial recovery. Treatment of optic nerve injury remains controversial.
